# Supplementary material for: Heterochromatin protein 1a functions for piRNA biogenesis predominantly from pericentric and telomeric regions in Drosophila
Source: Nat Commun. 2018 May 4;9:1735. doi: 10.1038/s41467-018-03908-3 (PMC5935673; doi:10.1038/s41467-018-03908-3)
Supplement: Supplementary file 1 — Supplementary Information [file 41467_2018_3908_MOESM1_ESM.pdf]

Supplementary information

Heterochromatin protein 1a functions for piRNA biogenesis predominantly from pericentric and telomeric regions in Drosophila.

Teo et al

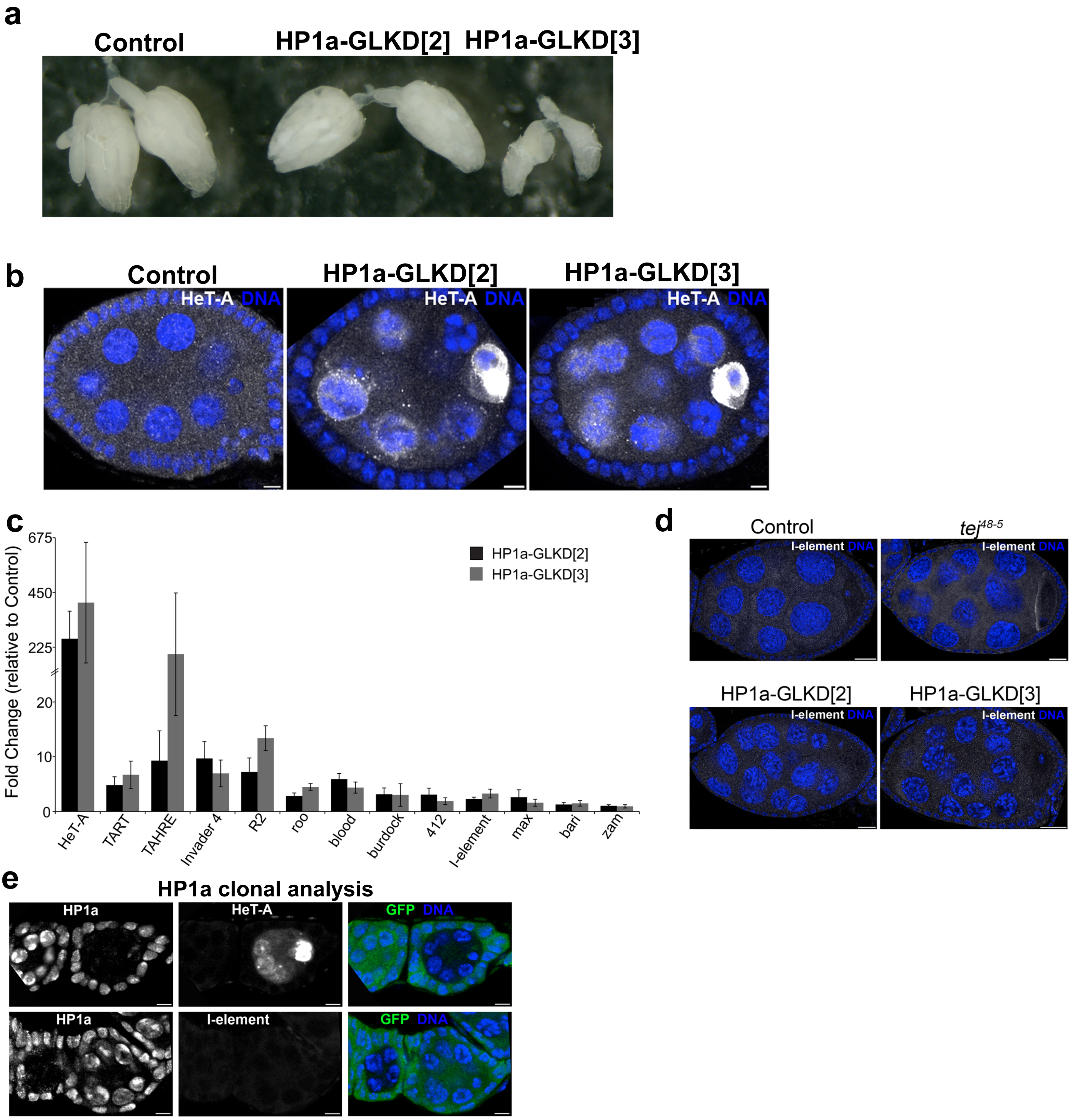

Supplementary Figure 1. Germline knockdown of HP1a leads to derepression of limited number of transposons. a Comparison of ovarian morphology from the sibling control and HP1a-GLKD[2] and HP1a-GLKD[3]. The HP1a-GLKD[3] ovaries appeared atrophic. n>10 independent experiments. b Confocal images representing immunostaining of HeT-A Gag protein immunostaining in the HP1a-GLKD and the control egg chambers. Scale bars represent 5 µm. n>10 independent experiments. c Barplot representing fold up-regulation of transposon families detected by qRT-PCR in HP1a-GLKD ovaries in comparison to the controls. Error bars represent standard error calculated from three biological replicates. n=3 independent experiments. d Immunostaining of I-element Gag protein in the HP1a-GLKD and tej^{48-5} egg chambers. Scale bars represent 20 µm. n=3 independent experiments. e Immunostaining of HP1a, HeT-A and I-element Gag proteins in germline clones of HP1a-null cells. HP1a mitotic clones are marked by the absence of GFP staining. Scale bars represent 10 µm. n=3 independent experiments.

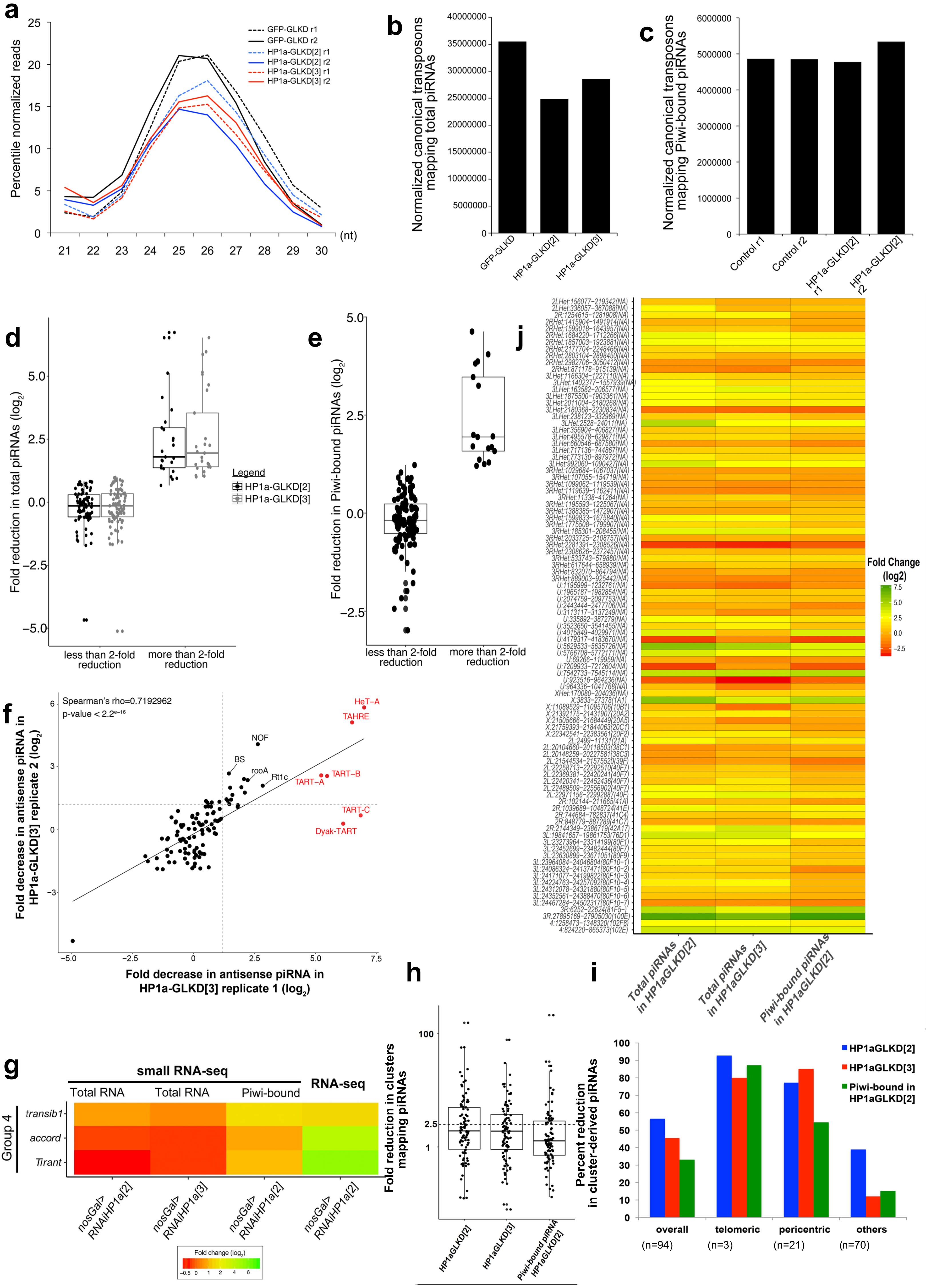

Supplementary Figure 2. HP1a loss causes reduction in piRNAs mapping to selective transposons. a Representation of size-wise abundance of small RNAs mapping to canonical transposons in the control GFP-GLKD and HP1a-GLKD ovaries. Data from all replicates of RNAi lines used in this study are shown. b Comparison of 23~29-nt total small RNA reads mapping to transposons in sense and antisense orientation between the control and HP1a-GLKD ovaries. c Comparison in levels of 23~29-nt Piwi-bound small RNAs mapping to canonical transposons between the control and HP1a-GLKD ovaries. d, e Boxplot showing fold reduction of total piRNA (d), and that of Piwi-bound piRNAs (e), mapping to transposon families in HP1a-GLKD ovaries compared to the control. n=2 independent experiments. f Comparison of reduction in total canonical-transposon mapping piRNAs among two replicates of HP1a-GLKD[3] in comparison to the control ovaries. g Heatmap showing the fold-reduction in overall and Piwi-bound small RNAs mapping to selected canonical transposons, and the fold up-regulation of corresponding transposon transcripts. h Boxplot showing reduction in the total and Piwi-bound piRNAs mapping to clusters in the HP1a-GLKD ovaries. Each dot represents a piRNA cluster. i Fold reduction in total and Piwi-bound piRNAs mapping uniquely to three different categories of piRNA clusters in HP1a-GLKD compared to the control ovaries. The clusters in the cytolocations marking chromosome ends (represented as telomeric), centromeres (represented as pericentric), and those in the rest of genome (others). n=2 independent experiments. j Heatmap representing reduction in total and Piwi-bound piRNAs mapping uniquely to major piRNA clusters in HP1a-GLKD ovaries. n=2 independent experiments. Boxplots in d,e and h; the middle line represents median, box represents 25–75 percentile range; called inter quartile range (IQR); upper and lower whisker extend highest or lowest values till 1.5 * IQR, values above and below are outliers and plotted individually

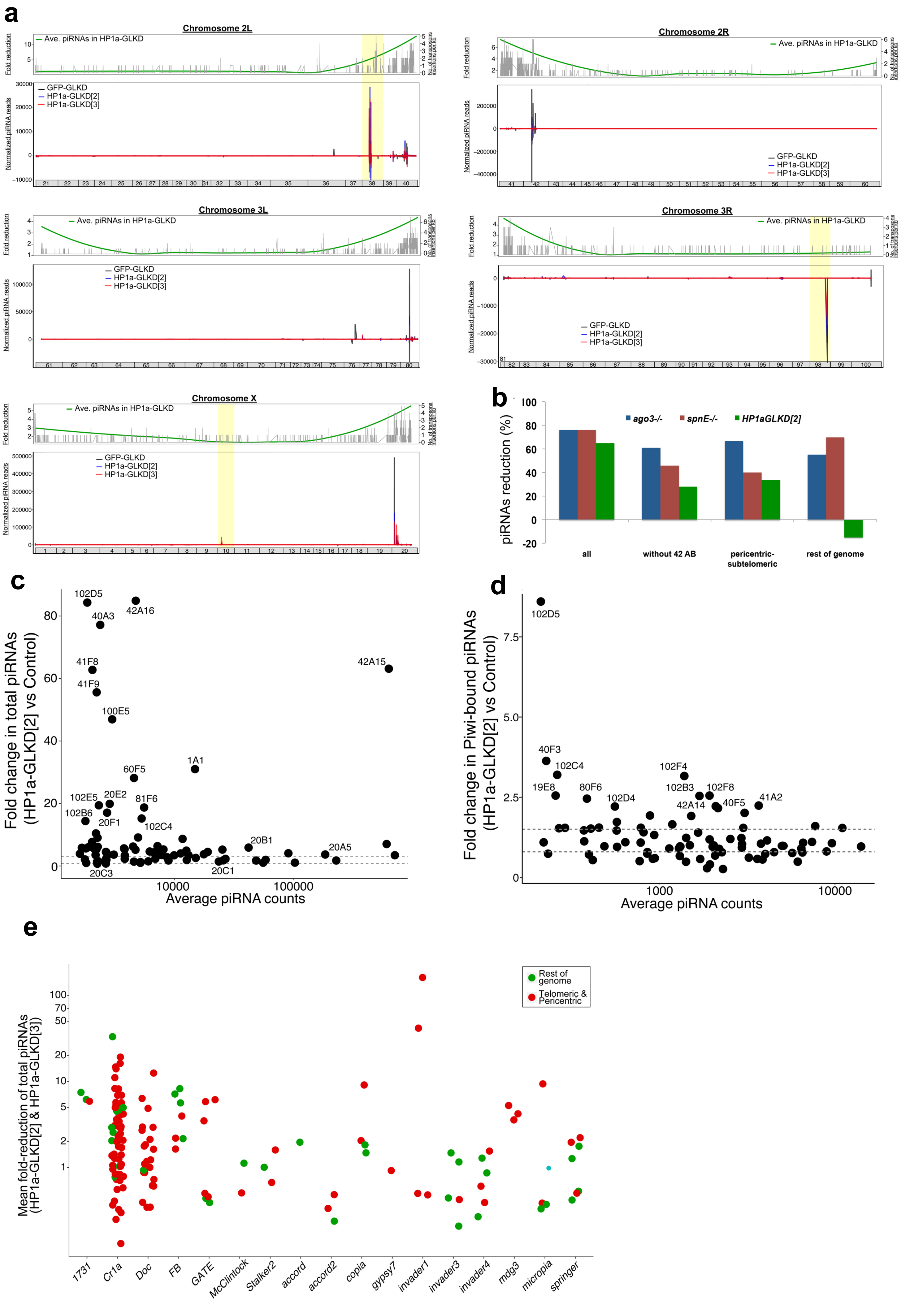

Supplementary Figure 3. HP1a loss causes a reduction in piRNAs mapping to selective transposons.
a Upper panels: Loess smoothening of fold reduction in the 23~29-nt uniquely mapping reads, in green, in 1-kb windows across all chromosome arms. The grey bars represent numbers of transposon insertions targeted by at least 10 unique-mappers, in each window. Lower panels: line plots of uniquely-mapping 23–29 nt reads on chromosome arms from HP1a-GLKD (blue and green) and the control ovaries (black). Regions highlighted in yellow correspond to selected piRNA hotspots, showing no effect of HP1aGLKD. b Barplots showing the reduction in piRNAs uniquely mapping to the transposon insertions in HP1a-GLKD and the piRNA pathway mutants, ago3, and spnE ovaries compared to their corresponding controls. We compared the piRNA loss arising from transposon insertions in cytolocations marking centromeres and telomeres, i.e. 1, 20, 21, 40, 41, 80, 100, 101, and 102 with that arising transposon insertions elsewhere in genome. c Reduction in total piRNAs mapping uniquely to transposon insertions in different cytolocations. The cytolocations, which had more than 1000 uniquely mapping total piRNAs to transposon insertions in control ovaries, were considered. n = 2 independent experiments. d Reduction in Piwi-bound piRNAs mapping uniquely to transposon insertions in different cytolocations. n=2 independent experiments. e Mean fold reduction in piRNAs mapping to each insertion of different transposon families that are derepressed in HP1a-GLKD replicates. Insertions with more than 1-kb in size and targeted by at least 20 unique-mappers were analysed. n=2 independent experiments.

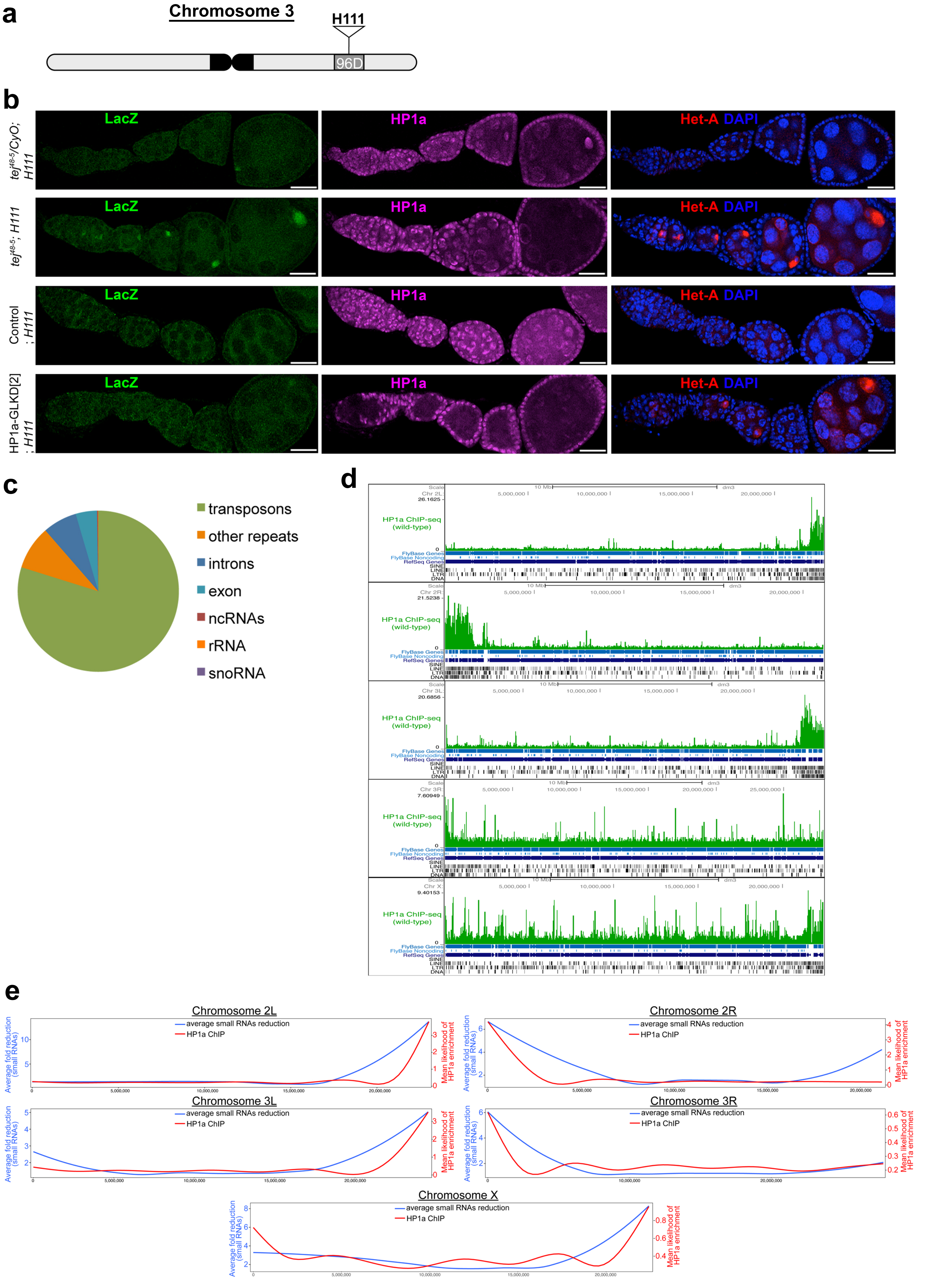

Supplementary Figure 4. Involvement of HP1a in the piRNA pathway is loci-dependent. a Schematic representing cytological position of another HeT-A/lacZ reporter used in this study. b Representative confocal image of immunostaining of HP1a (magenta), LacZ (green), and HeT-A-Gag (red) in tej48-5, tej48-5/CyO and HP1a-GLKD[2] ovaries containing the HeT-A/lazZ transgene (H111 line). Scale bars represent 25 µm. n=3 independent experiments. c Pie charts representing the distribution of HP1a ChIP-seq peaks mapping to transposons, exons, introns, rRNAs, snoRNAs, non-coding RNAs, and other repeats in wild-type ovaries. d UCSC browser screen-shot showing HP1a ChIP-seq reads across arms of chromosomes X, 2, and 3. Reads in wild-type ovaries were normalized to those in the IgG ChIP-seq library. e Blue line with the scale on the right axis: loess smoothening plots of the average reduction in the 23~29-nt mapping small RNA reads from HP1a-GLKD[2] and HP1a-GLKD[3] in comparison to the control ovaries. n=2 independent experiments. Red line with the scale on the left axis: the mean likelihood of HP1a enrichment after normalizing to reads in the IgG ChIP-seq library in 1-kb windows across all chromosome arms.

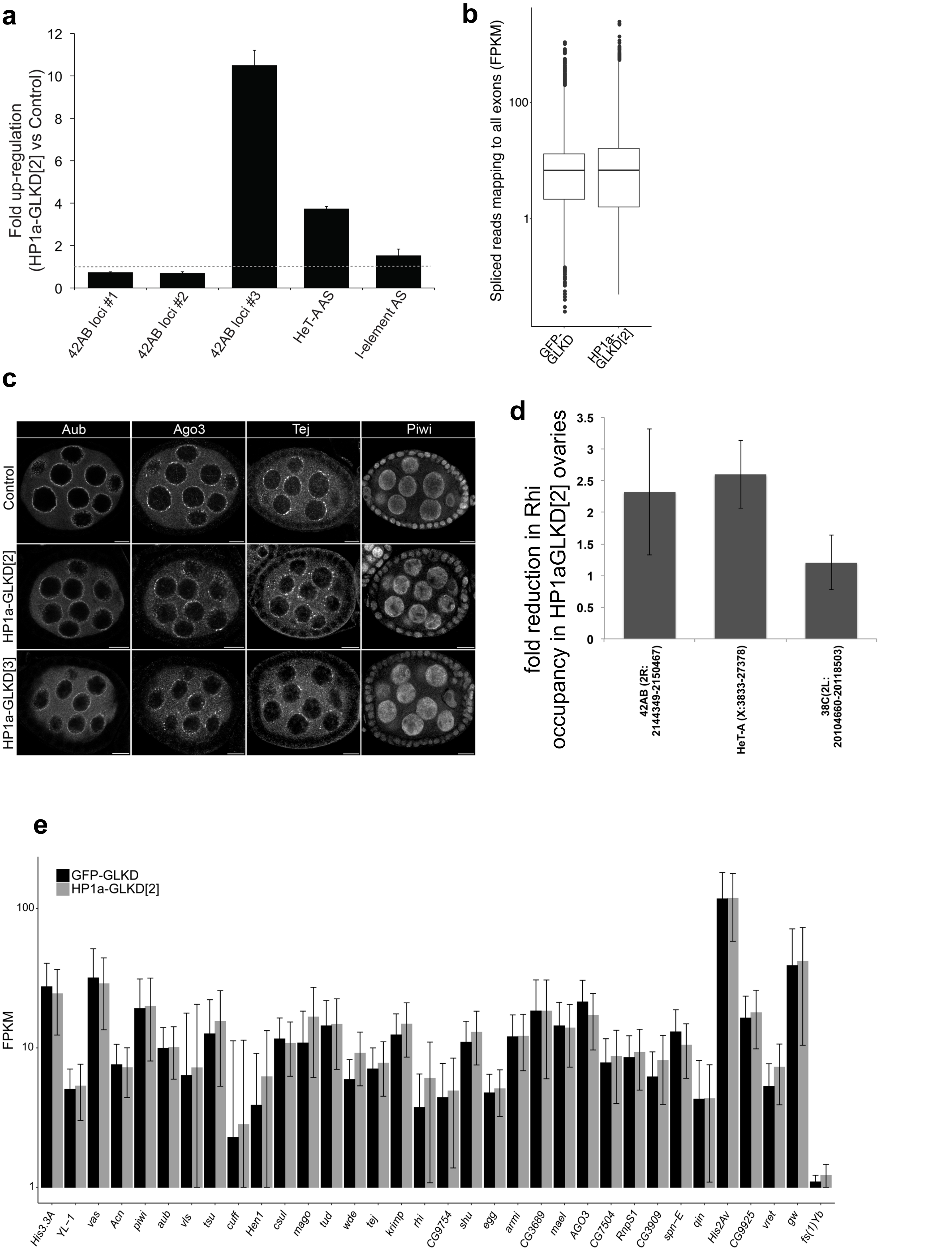

Supplementary Figure 5. HP1a loss affects the splicing of piRNA precursors. a Barplot representing fold change of strand-specific transcript levels detected by qRT-PCR in HP1a-GLKD ovaries in comparison to the control. Three regions in cluster 42AB, and the each antisense transcript (AS) originating from HeT-A and I-element transposon were examined. Error bars represent standard error calculated from three biological replicates. b Comparison of FPKM values for spliced exon-mapping transcripts between control and HP1a-GLKD ovaries. . The middle line represents median, box represents 25–75 percentile range; called inter quartile range (IQR); upper and lower whisker extend highest or lowest values till 1.5 * IQR, values above and below are outliers and plotted individually. c Representative confocal images of immunostaining of piRNA pathway components Aub, Ago3, Tej, and Piwi in HP1a-GLKD and control ovaries. Scale bars represent 10 µm. n=3 independent experiments. d ChIP-qPCR showing the changes in the Rhi occupancy in HP1a-GLKD[2] ovaries at clusters *42AB, 1A* and *38C*, Two biological replicates were analysed for Rhi binding in above genotypes. e Comparison of steady state transcript levels of the known piRNA pathway components in HP1a-GLKD[2] and the control GFP-GLKD ovaries.

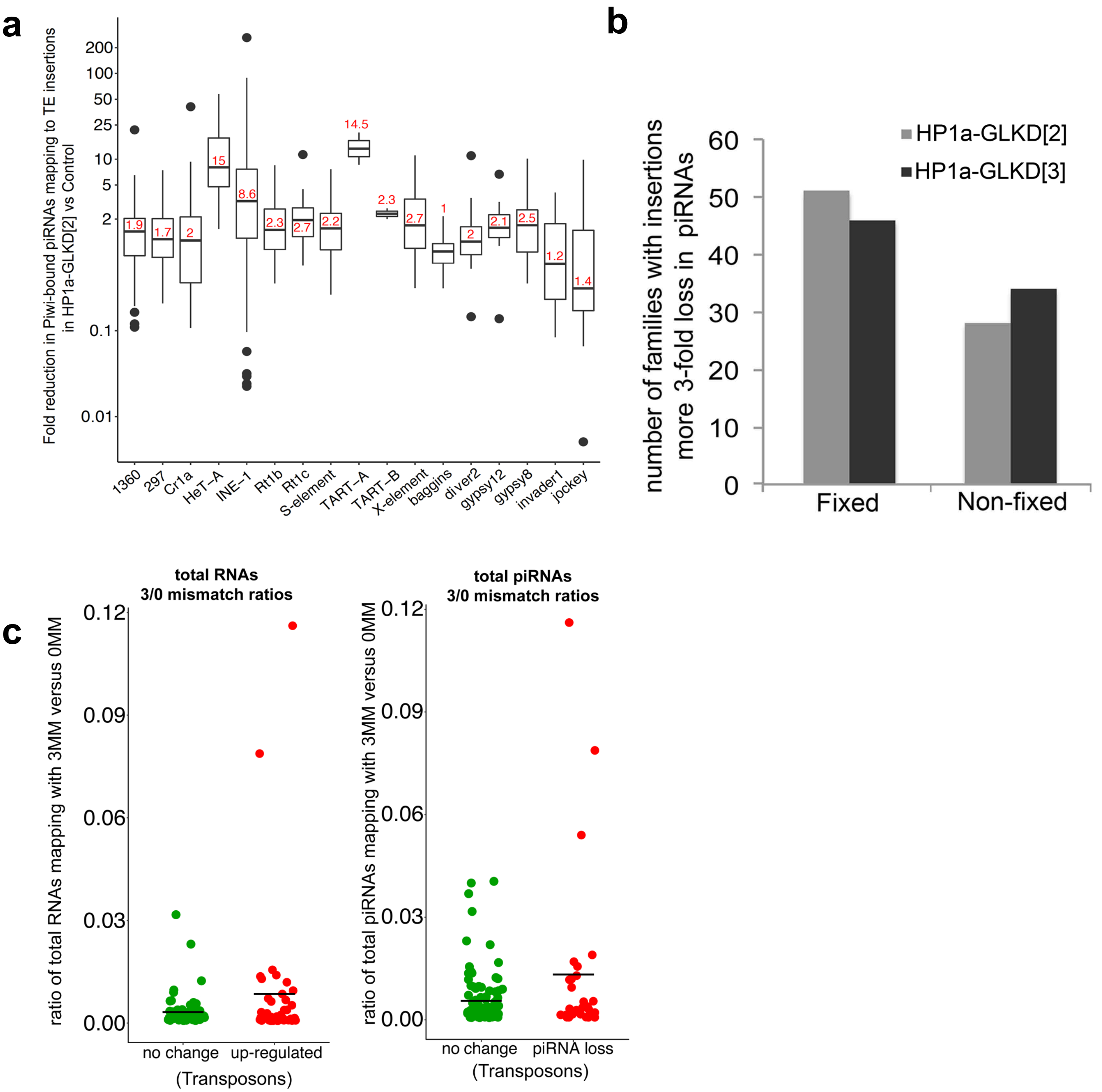

Supplementary Figure 6. HP1a is primarily required for piRNA targeting evolutionarily old transposons. a Boxplot showing the reduction in the Piwi-bound piRNAs uniquely mapping to the insertions of transposon families, with frequent insertions, in HP1a-GLKD ovaries. Mean fold change of two biological replicates is shown. . The middle line represents median, box represents 25–75 percentile range; called inter quartile range (IQR); upper and lower whisker extend highest or lowest values till 1.5 * IQR, values above and below are outliers and plotted individually. b Comparison of piRNA loss for fixed and non-fixed insertions across all transposon families in HP1a-GLKD ovaries compared to the control. c Scatter plots showing three-to-zero-mismatch ratios of the piRNAs mapping to transposons families. Left: for transposon families showing no upregulation (green dots) and those showing more than 2-fold upregulation in HP1a-GLKD ovaries, compared to control ovaries. Right: transposon families showing no reduction in piRNAs (green dots) and those with more than 2-fold reduction in piRNAs (red dots) upon HP1a-GLKD. n=2 independent experiments.
