# Supplementary material for: Heterochromatin protein 1a functions for piRNA biogenesis predominantly from pericentric and telomeric regions in Drosophila
Source: Nat Commun. 2018 May 4;9:1735. doi: 10.1038/s41467-018-03908-3 (PMC5935673; doi:10.1038/s41467-018-03908-3)
Supplement: Supplementary file 3 — Description of Additional Supplementary Files [file 41467_2018_3908_MOESM3_ESM.pdf]

Description of Additional Supplementary Files

File Name: Supplementary Data 1
Description: Information about the libraries used in the study.

File Name: Supplementary Data 2
Description: Fold upregulation of canonical transposons in HP1aGLKD[2] ovaries in comparison to sibling control: RNA-seq data was aligned to canonical transposons and fold increase in FPKM values in HP1aGLKD[2] ovaries is represented.

File Name: Supplementary Data 3
Description: Reduction in the total and Piwi-bound piRNAs mapping to canonical transposon families in HP1aGLKD ovaries.

File Name: Supplementary Data 4
Description: Reduction in the total and Piwi-bound piRNAs uniquely mapping to piRNA clusters in HP1aGLKD ovaries.

File Name: Supplementary Data 5
Description: Average number of piRNAs (unique and mutimapper) mapping to insertions belonging to different transposon families in *D. melanogaster* in control ovaries.

File Name: Supplementary Data 6
Description: List of primers used in the study.
